# Noisy visual feedback training impairs detection of self-generated movement error: implications for anosognosia for hemiplegia

**DOI:** 10.3389/fnhum.2014.00456

**Published:** 2014-06-24

**Authors:** Catherine Preston, Roger Newport

**Affiliations:** ^1^Brain, Body and Self Laboratory, Department of Neuroscience, Karolinska Institutet, StockholmSweden; ^2^University of Nottingham, NottinghamUK

**Keywords:** agency, motor awareness, anosognosia for hemiplegia, comparator model, forward models

## Abstract

Anosognosia for hemiplegia (AHP) is characterized as a disorder in which patients are unaware of their contralateral motor deficit. Many current theories for unawareness in AHP are based on comparator model accounts of the normal experience of agency. According to such models, while small mismatches between predicted and actual feedback allow unconscious fine-tuning of normal actions, mismatches that surpass an inherent threshold reach conscious awareness and inform judgments of agency (whether a given movement is produced by the self or another agent). This theory depends on a threshold for consciousness that is greater than the intrinsic noise in the system to reduce the occurrence of incorrect rejections of self-generated movements and maintain a fluid experience of agency. Pathological increases to this threshold could account for reduced motor awareness following brain injury, including AHP. The current experiment tested this hypothesis in healthy controls by exposing them to training in which noise was applied the visual feedback of their normal reaches. Subsequent self/other attribution tasks without noise revealed a decrease in the ability to detect manipulated (other) feedback compared to training without noise. This suggests a slackening of awareness thresholds in the comparator model that may help to explain clinical observations of decreased action awareness following stroke.

## INTRODUCTION

Under normal circumstances we have no difficulty in recognizing our own movements and knowing when we have, or have not, performed an action. However, this can be disrupted following brain injury or stroke. One such disorder that has been well described is anosognosia for hemiplegia (AHP) and is characterized as a disorder, normally following right hemisphere stroke, in which the patient is not aware of their contralateral (left) motor deficit (Jenkinson et al., 2011). Such patients claim to be able to perform actions normally despite their obvious paralysis, even to the extent that when asked to execute an action some can claim to be doing so when their limb is motionless (paralyzed) at their side (Ramachandran, 1996).

Most of the current theories explaining AHP focus on forward models originally described to explain motor control. These forward models incorporate comparators, which compare motor commands and intentions with actual and predicted sensory feedback. Normally the errors detected are small and do not reach conscious awareness but allow the motor system to correct and fine-tune our movements (Miall and Wolpert, 1996; Wolpert, 1997). Another role ascribed to the comparators is for the discrimination between *Self* and *Other* generated actions (agency attribution). When the discrepancies detected at the comparators are large they reach conscious awareness and influence the experience of agency (the feeling of causation over an action). There is a general consensus within theories based on the comparator model that in AHP, erroneous feelings of agency over actions that are never executed are based solely on comparisons between intact intentions and motor predictions. Such that these patients retain the ability to form motor intentions and produce an efference copy of the action on which a prediction of the next state of the motor system is formed. However, due to their paralysis AHP patients never actually initiate the action. With normal functioning of the comparators, the lack of movement from the paralyzed limb would highlight large discrepancies with that intended or predicted, thus informing the individual of their paralysis. However, AHP patients do not appear to detect these discrepancies and thus remain unaware of their motor deficit.

Exactly why these large discrepancies do not reach conscious awareness is as yet unclear. Frith et al. (2000) suggested that these discrepancies are ignored, which may in part be due visual neglect that is frequently a co-morbid deficit of AHP. This explanation cannot fully account for this; however, given that double dissociations of neglect and AHP have been identified (Bisiach et al., 1986; Jehkonen et al., 2006). Other explanations for ignoring these discrepancies are not fully described and so difficult to test experimentally. Berti and Pia (2006) suggested that, although the rest of the comparator functions normally, the comparators monitoring sensory feedback are broken. Although this explains why the inability to produce movement is not detected, if the comparators are destroyed it does not adequately account for reinstatement of awareness, which commonly occurs in AHP after a few weeks (Jenkinson et al., 2011). Recently, a third hypothesis was put forward suggesting that these comparators, rather than being broken, have pathological slackening of awareness thresholds (Preston et al., 2010). As stated above, most discrepancies detected by the comparator model are used for fine-tuning movements and do not reach conscious awareness and as such it is logical to assume that there is a threshold that needs to be reached in order to penetrate consciousness. It is also logical to assume that any threshold should be greater than the inherent noise in the system, a threshold, which is likely to be seriously increased following brain damage. It was thus suggested that in AHP the threshold is pathologically increased to the extent that all movements, and indeed no movement at all, do not reach threshold and so are accepted as successful *Self* produced actions.

Preston et al. (2010) found support for this theory from a single AHP patient, GG. Interestingly it was found that GG, in addition to a lack of awareness for his left sided paralysis, was also unaware of actions produced with his intact (right) arm [an observation that had only previously been reported anecdotally, Ramachandran (1995)]. This allowed experimental investigation of comparator functioning of a moving limb in terms of low-level motor control as well as high-level awareness of action. It was found that, following large spatial perturbations being applied to visual feedback of his right handed reaching movements, GG was able to make crude motor corrections to his reaches in an attempt to compensate for the visual perturbation, whilst remaining unaware of large inaccuracies in his movements, any corrective movements he was making (including large secondary movements), or that any such perturbations were applied. A control sample of hemiplegic neglect patients without AHP did perform worse than young healthy controls, but were able to detect some larger perturbations (unlike GG who never reported being aware). The fact that GG was able to make some corrections to his movements, albeit poorly, implies that the comparators are working to an extent and thus arguing against broken comparators as suggested by Berti and Pia (2006). However, as these motor corrections never reached consciousness, such findings are in line with a slackening of comparator thresholds – something that was observable to an extent in the neglect control group, but was extreme in the AHP patient. However, this was based on observations of a single AHP patient and a small control group so further research is clearly needed.

The aim of the current study was to further test the threshold theory of AHP using neurologically intact controls. If comparator thresholds of motor awareness are governed by inherent noise in the system (i.e., the threshold should be at least as great as the noise) increasing noise to feedback of movements made by healthy controls should serve to increase thresholds and so leading them to accept greater discrepancies between their actual movement and the visual feedback as true representations of their actions.

An important factor found to inform judgments of agency involves conscious motor intention, such that you are more likely to attribute an observed action as self-generated if it accurately attains your intended movement goal (e.g., accurately reaches the target). Systematic visual distortions applied across a series of reaches can induce motor learning such that adjustments are made to the motor commands in order to compensate for the distortions and maintain accuracy of the reach (e.g., Izawa and Shadmehr, 2011). Through such paradigms, dissociations between low-level motor planning and high-level motor awareness have been demonstrated. Gradually increasing systematic distortions to visual feedback of reaches produces gradual changes in reach trajectory without conscious awareness to the extent that when shown veridical feedback of the actual reach participants deny that it is a true representation of their action (Synofzik et al., 2006; Preston and Newport, 2010). Thus visual perturbations applied to feedback of reaches can modulate conscious error detection (agency) through changes to reach trajectory via unconscious (sub threshold) motor correction mechanisms. Distortions applied to the visual feedback of reaches that, rather than being systematic, are randomly selected from a distribution leads to learning the mean of that distribution (Scheidt et al., 2001). Therefore if the mean of the distribution is veridical feedback (no perturbation), there should be no effect on reach accuracy as participants retain highest accuracy for unperturbed reaches. Therefore any changes to observed conscious error detection as a result should not be an indirect effect of reach accuracy, but a direct modulation of conscious awareness thresholds.

Participants received visual feedback of reaching movements using a vBOT robotic manipulandum. All participants took part in a self/other detection task similar to that described in Preston et al. (2010). This was completed after both noise and no-noise training with each participant. It was predicted that the percentage *Self* judgments to visually perturbed trials in the detection task would increase following noise training compared to following no-noise training, without any significant effect on reach accuracy.

## MATERIALS AND METHODS

### PARTICIPANTS

Twenty-two neurologically healthy participants (seven males) took part in the experiment with a median age of 20 years (range 20–55 years). All were right hand handed and had normal or corrected to normal vision. The experiment was conducted in accordance with the local ethics committee and the declaration of Helsinki.

### MATERIALS

**FIGURE 1:**
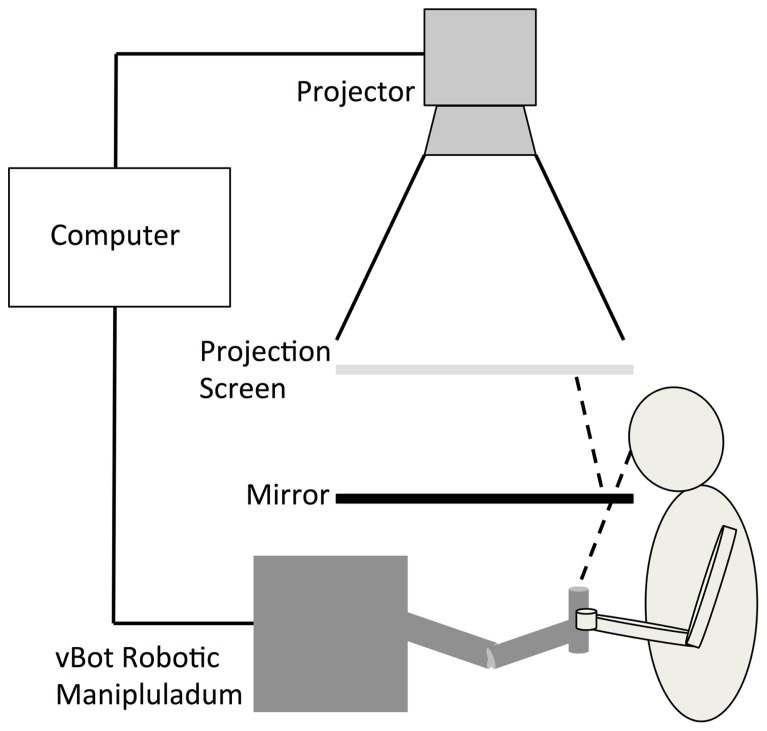
**The experimental set-up was similar to that described in Preston et al. (2010).** Participants viewed the cursor via a horizontal mirror that was positioned equidistant between the limb and projection screen such that visual feedback of their movements appeared in the same spatial plane as the actual reaching limb.

Participants’ reaches were represented by the movements of a white cursor (20 mm in diameter) that was projected, along with the target location, onto a horizontal semi-transparent screen positioned 450 mm above the reaching limb. Participants viewed the cursor via a horizontal mirror that was positioned equidistant between the limb and projection such that visual feedback of their movements appeared in the same spatial plane as the actual reaching limb (see **Figure 1**). The location of the cursor was calculated on-line using position data recorded by a vBOT 2D robotic manipulandum sampling at 1000 Hz (see Howard et al., 2009 for a comprehensive description of this device).

### PROCEDURE

**FIGURE 2:**
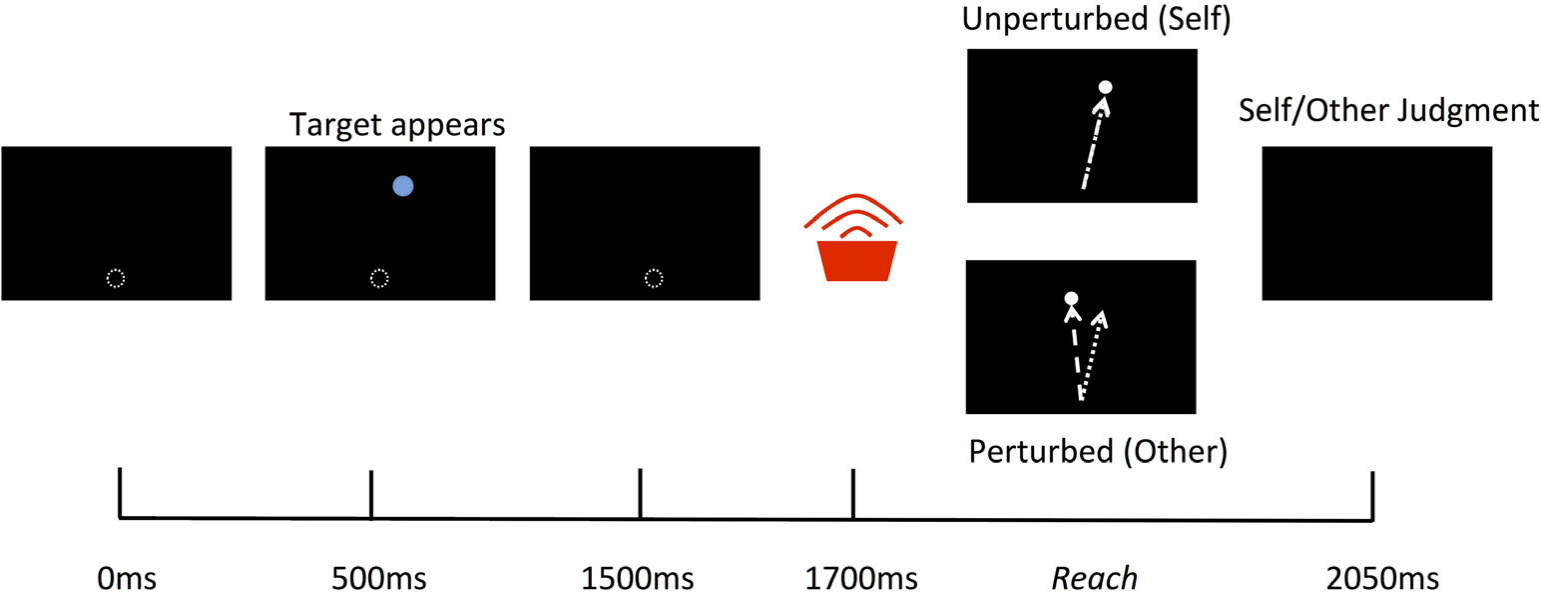
**Schematic of a single trial.** 500 ms after the vBOT moved the limb to the start location a target appeared for 1000 ms followed by a tone indicating for participants to begin their reach. A white cursor represented the movements of the real limb and was either an exact representation of the actual movement (*Self*) or had a spatial perturbation applied (*Other*). At the end of the reach participants were required to give a verbal forced choice judgment as to whether the observed movement was that of *Self* or *Other.*

Participants sat looking down into the mirror and held onto the vBOT handle with their right hand. Before the beginning of each trial the vBOT moved the limb to a start location just out of view and directly in front of the body midline then there was a 500 ms delay before the trail commenced. At the beginning of each trial a blue circular target with a diameter of 30 mm appeared for 1000 ms at randomly varying locations on the screen averaging 210 mm forward from the start location and directly inline with the start position. 200 ms following the disappearance of the target a tone sounded to indicate that the participants should begin their reach. The participants then had 1250 ms to complete their reach before the cursor disappeared and the vBOT move the limb back to the start location (see **Figure 2**). Visual feedback of the reaching movements was represented by the movements of the white cursor and was either an exact representation of their actual movement (*Self*) or had an angular perturbation applied (*Other*), for which the angle of the cursor trajectory was rotated relative to the actual reach trajectory by varying degrees. *Other* actions were defined as actions under the control of the computer (i.e., not the same as the movement performed), as opposed to the actions of another human being. This was made clear to, and understood by, all participants prior to the experiment.

The experiment contained two conditions, *Noise* and *No-Noise*, the order of which was counterbalanced between participants. For each condition participants first completed a training block, in which the experimental variable was modulated (and which differed between conditions) followed by a judgment block, which was identical for both conditions. Training blocks consisted of 80 trials in which participants were required to execute reaching movements whilst instructed only to be as accurate as possible to the target. For the *Noise* condition the visual feedback in the training block was equally divided between –2^∘^, –4^∘^, –6^∘^, –8^∘^, –10^∘^, 2^∘^, 4^∘^, 6^∘^, 8^∘^, and 10^∘^ perturbations (eight trials per perturbation size, with negative values indicating leftward perturbations. Degrees of perturbation refers to the angle between the start and end point of the cursor trajectory relative to the actual reach trajectory). In the *No-Noise* condition, all reaches were veridical to the actual movements. Judgment blocks consisted of 56 trials equally divided between –12^∘^, –8^∘^, –4^∘^, 0^∘^, 4^∘^, 8^∘^, and 12^∘^ perturbations (eight per perturbation size). Following each reach, participants were required to make a forced choice verbal judgment as to whether the visual feedback had been controlled by themselves (*Self*) or by the computer (*Other*). Only trials in which the participant failed to initiate a reach within the time window were rejected (<2% of total trials).

Prior to the experimental conditions, participants took part in three practice blocks in order to familiarized them with the vBOT, the timing of the reaching movements, and what was meant by *Other* visual feedback. The first practice block contained 10 trials of only veridical visual feedback (*Self* trials). For this block participants were informed that all visual feedback was an exact representation of their actual reaches and so were not required to give self/other judgments. The second practice block also consisted of 10 trials but with perturbation sizes of 0^∘^, 10^∘^, –10^∘^, 20^∘^ and –20^∘^ (two trails per perturbation). Participants were required to make a self/other judgment at the end of each trial as in the judgment blocks. The third practice block consisted of 56 trials and was identical to the judgment block described above. The trial order in all the individual blocks was randomized.

## RESULTS

### SUBJECTIVE JUDGMENTS

The self/other judgment data were converted into a percentage *Self* score for each perturbation size (collapsed across left/right direction) and entered in a 2 × 4 repeated measures ANOVA with the factors condition (*Noise, No-Noise*) and perturbation (0^∘^, 4^∘^, 8^∘^, 12^∘^).

**FIGURE 3:**
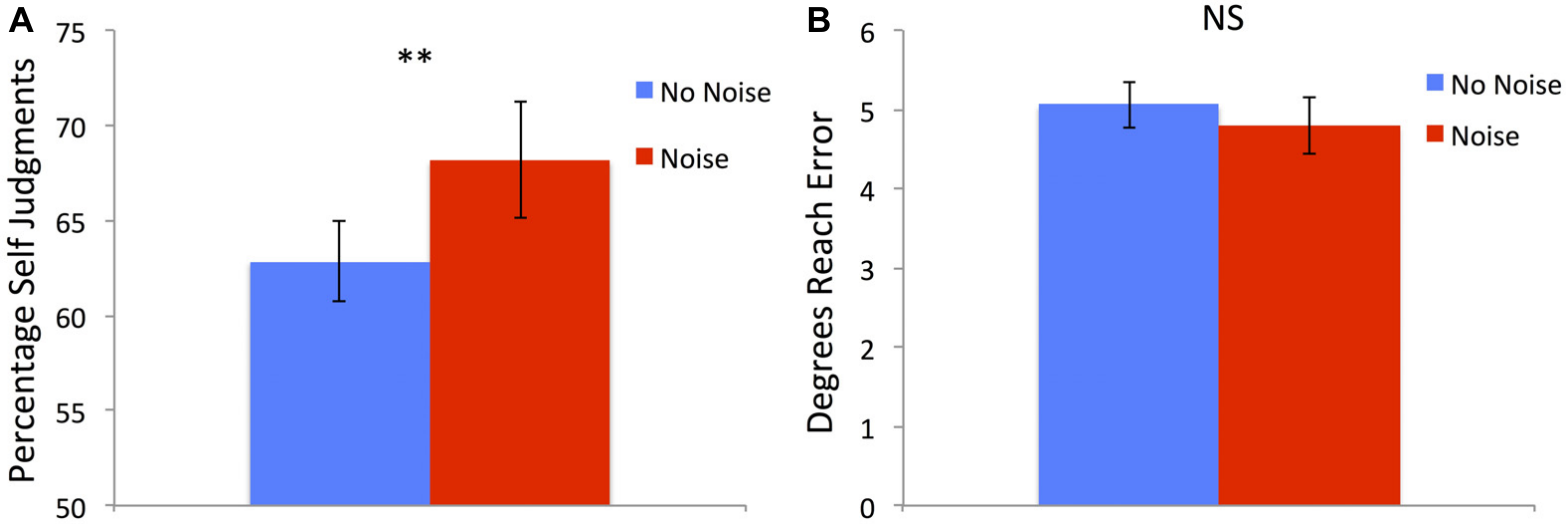
**(A)** Percentage of *Self* judgments was significantly higher following *Noise* training compared to *No-Noise* training across all perturbation sizes.**(B)** Absolute degrees of end point reach error did not differ between the different training conditions. Error bars show SE and ** denotes significant difference < 0.01.

There was a significant main effect of condition [*F*(1,21) = 8.69, *p* = 0.008] with the *Noise* condition having a higher percentage of *Self* judgments (mean = 68.71%, SD = 14.24%) compared to the *No-Noise* condition (mean = 62.86%, SD = 9.9%; see **Figure 3A**). There was also a main effect of perturbation [*F*(3,63) = 145.89, *p* < 0.001] with 0^∘^ having the greatest percentage of *Self* responses (mean = 92.77, SD = 9.81), followed by 4^∘^ (mean = 85.88%, SD = 12.86%), 8^∘^ (mean = 54.91%, SD = 16.7%) then 12^∘^ (mean = 28.49%, SD = 19.71%). There was no significant interaction [*F*(3,57) = 1.19, *p* = 0.321].

### REACHING ACCURACY

Mean endpoint and midpoint errors were calculated for reaches in the judgment blocks for both training and no-training conditions. Errors were calculated as the angle in degrees between a straight line from the start point to the target and from the start point to the cursor position at the end or midpoint of the reach for endpoint and midpoint errors, respectively. Because of the inclusion of both left and right sided perturbations, endpoint errors were calculated as absolute values and then entered in separate 2 × 4 repeated measures ANOVAs with the factors condition (*Noise, No-Noise*) and perturbation (0, 4, 8 and 12^∘^).

### ENDPOINT ERROR

There was a significant main effect of perturbation [*F*(3,63) = 204.8, *p* < 0.001] with 0^∘^ having the smallest errors (mean = 2.56^∘^, SD = 1.15^∘^), followed by 4^∘^ (mean = 3.31^∘^, SD = 1.13^∘^), 8^∘^ (mean = 5.44, SD = 1.73) then 12^∘^ (mean = 8.4^∘^, SD = 2.29^∘^). Importantly there was no significant effect of training condition [*F*(1,21) = 1.7, *p* = 0.206; see **Figure 3B**] and there was also no significant interaction [*F*(3,57) = 2.03, *p* = 0.121].

### MIDPOINT ERROR

There was a significant main effect of perturbation [*F*(3,63) = 334, *p* < 0.001] with 0^∘^ having the smallest errors (mean = 3.93^∘^, SD = 1.48^∘^), followed by 4^∘^ (mean = 4.95^∘^, SD = 1.02^∘^), 8^∘^ (mean = 7.94^∘^, SD = 0.756^∘^) then 12^∘^ (mean = 11.25^∘^, SD = 0.698^∘^). There was no significant effect of training condition [*F*(1,21) = 0.037, *p* = 0.849] and there was no significant interaction [*F*(3,57) = 0.665, *p* = 0.577].

## DISCUSSION

The current results demonstrate that following a period of training in which noise was added to visual feedback, participants were less able to perceive perturbations to their movements on a subsequent detection task: that is, a greater number of trials were judged by participants as being controlled by themselves (*Self*) across all perturbations. This suggests that the threshold at which we become consciously aware of discrepancies between our actions and sensory feedback can be increased by introducing noise to the motor system. It has long been suggested that comparator based forward models of motor control have a threshold below which discrepancies detected by the system do not reach consciousness (Frith et al., 2000). Moreover, the fact that we are largely unaware of small corrections to our actions has been consistently demonstrated using different paradigms (e.g., Goodale et al., 1986; Fourneret and Jeannerod, 1998), but this is the first demonstration that the level of unawareness can also be increased at will.

The current data lends support to the threshold theory as an explanation for AHP. Within current explanations of AHP based on forward model comparator systems there is a general agreement that awareness of action in these patients is dictated by motor predictions rather than sensory feedback. Due to limited experimental evidence there is disagreement as to why such large discrepancies caused by hemiplegia go undetected by consciousness awareness. The threshold theory suggests that such unawareness occurs due to a pathological slackening of the normal comparator thresholds. Here, it has been demonstrated in neurologically intact participants, that the threshold at which a visual/motor mismatch reaches conscious awareness can be broadened by experimentally increasing noise of visual feedback. This therefore demonstrates that consciousness thresholds in the motor system can be manipulated and hence it is plausible that this normal adaptability of thresholds can be pathologically increased following extensive brain damage.

In terms of implicated brain regions, AHP does not have a clear-cut pathology. Unawareness for left sided hemiplegia has been associated with larger lesion sizes (Orfei et al., 2007) as well as numerous co-morbid deficits (although none have be found to fully account for AHP symptomology; Jehkonen et al., 2006). Due to these factors it is unsurprising that various brain areas have been identified in AHP pathology, including frontal parietal networks (Pia et al., 2004), premotor areas (Berti et al., 2005) and the insula cortex (Berti et al., 2005; Karnath et al., 2005; Vocat et al., 2010). Although evidence from healthy controls places the comparator in the right parietal lobe (Farrer et al., 2003; Preston and Newport, 2008), all of these brain areas have been independently associated with motor control and/ or action awareness (e.g., Haggard and Magno, 1999; Farrer and Frith, 2002; Farrer et al., 2003). If AHP is caused by a pathological increase in awareness thresholds due to increased inherent noise, this could be a result of damage to multiple sites associated with action planning and execution and not just regions specifically involved with the comparator. Therefore, extensive lesion sites covering various combinations of motor related regions, as are associated with AHP, may feasibly result in a greater increase in noise throughout the entire system, explaining why no single brain area has been uniformly identified in the etiology of AHP.

Importantly there was no effect of the noise training on reach accuracy. Previously it has been suggested that goal attainment and motor intention (accurately reaching the target) are strong predictors for judgments/feelings of agency (Farrer et al., 2008; Preston and Newport, 2010). Indeed post hoc analysis of reach accuracy for perturbed trails judged as *Self* vs. those judged to be *Other*, find the former to be significantly more accurate for both training [*t*(21) = 6.19, *p* < 0.001] and non-training [*t*(21) = 6.57, *p* < 0.001] conditions. However, due to the lack of difference in accuracy between the conditions, the observed increase in *Self* judgments following noise training cannot be explained by participants being more accurate to the target. This suggests that following noise training, participants accepted larger reach errors as accurate representations of their own movements; in other words a general broadening of what is accepted as *Self*.

Other implications of these results include the interpretation of agency and movement recognition experiments. Experimental paradigms that include numerous different perturbation sizes over the same or several consecutive blocks, by their very nature increase noise in the visual feedback and so are likely to result in poorer detection of discrepancies. Similarly, when fewer different discrepancies are used, detection may become more sensitive. For example, Preston and Newport (2008) report fewer than 50% *Self* judgments for perturbations of 4^∘^ when only presenting feedback of 0^∘^ and 4^∘^. In the current experiment, however, which uses a greater number of perturbation sizes, the mean percentage of *Self* judgments to a 4^∘^ perturbation is over 80%, even before noise training. Moreover, these values are also different to those observed by Farrer and colleagues (Farrer et al., 2003, 2008) when using a broad range of perturbations, but with shorter reach distances (resulting in relatively smaller end-point errors). Caution must be applied, therefore, when comparing across different experimental paradigms.

A possible limitation of the current study concerns implementation of motor correction during reaching. Because the cursor was visible throughout the reach, low-level (unconscious) motor correction mechanisms could have been recruited that maintained accuracy to the target despite the visual perturbations – thereby influencing self/other judgments. While mean midpoint errors were larger than endpoint errors, this is to be expected due the curvature observed in normal reaching. It should also be noted that any online corrections that may have occurred were incomplete as both mid and endpoint errors increased with perturbation size. Moreover, any correction that did occur was equivalent for both conditions as accuracy at mid and endpoint were not significantly different between training and no-training conditions. Future studies, however, could further deconstruct the mechanisms of low and high level processes in motor awareness by modulating the visibility of the cursor at the beginning and end of the reach. Another consideration for future studies is to vary the range of noise applied in the training blocks. In the current study a smaller range of noise was applied in the training compared to test blocks. This meant that although the awareness threshold for error detection was expanded it still followed the same pattern as under normal reaching conditions, such that noise inherent in the system (training block) would be smaller than the range of errors that could occur during every day reaching (test block). However, with AHP it is suggested that the thresholds are expanded beyond the inherent noise so that no perturbations are consciously perceived. Future studies could investigate the effect of larger noise ranges in the training blocks relative to test blocks so as to be more comparable to AHP.

A further point of interest for future research is the role of oculomotor strategies. Previous studies have shown that eye movements can effect perception of limb movements (Ariff et al., 2002; Scherberger et al., 2003). However, to date little is known about eye movements during agency attribution tasks or during action (and attempted action) in AHP patients. Monitoring eye movements during tasks such as that described in the current study with both healthy and brain damaged participants may help shed light on the role of eye movements for action awareness and how this might be effected by noise training.

In conclusion, the current study demonstrates that exposing neurologically intact participants to noisy visual feedback can reduce their ability to consciously detect visual discrepancies applied to the feedback of their actions in a subsequent self/other action recognition task. This provides support for the threshold theory of AHP, which suggests that the disorder may be caused by pathological slackening of comparator thresholds within the motor system. These data also have implications concerning experimental models of action awareness given that awareness thresholds can be so easily manipulated by experimental design.

## AUTHOR CONTRIBUTIONS

Both authors contributed equally to the design of the experiment and the interpretation of the data. Catherine Preston conducted the data analysis and wrote the initial draft, which was iteratively edited by Roger Newport and Catherine Preston. Both authors approved the final version of the manuscript and are fully accountable for the work.

## Conflict of Interest Statement

The authors declare that the research was conducted in the absence of any commercial or financial relationships that could be construed as a potential conflict of interest.
